# CRISPRi Induced Suppression of Fimbriae Gene (*fimH*) of a Uropathogenic *Escherichia coli*: An Approach to Inhibit Microbial Biofilms

**DOI:** 10.3389/fimmu.2017.01552

**Published:** 2017-11-13

**Authors:** Azna Zuberi, Nayeem Ahmad, Asad U. Khan

**Affiliations:** ^1^Medical Microbiology and Molecular Biology Laboratory, Interdisciplinary Biotechnology Unit, Aligarh Muslim University, Aligarh, India

**Keywords:** biofilm, CRISPRi, fimH, bacteria, immunity, gene, fimbriae

## Abstract

Urinary tract infection (UTI) is one the common infections caused by the recalcitrant nature of biofilms, developed after the pathogen has adhered to the inner lining of the urinary tract. Although significant research has been made in recent years to control these types of infection, but as of yet, no approach has sufficiently been able to reduce the prevalence of UTIs. The main objective of this study was to prevent UTIs through targeting the fimH gene, which is the major virulent factor responsible for biofilm formation. The novelty of this work lies in the use of CRISPRi, a gene specific editing tool to control such types of infections. Accordingly, the system was designed to target *fimH* gene, responsible for bacterial adherence and this approach was successfully validated by performing microscopic, biofilm and adherence assays.

## Introduction

Urinary tract infection (UTI) is considered as one of the most important causes of health issues and morbidity, worldwide, affecting persons of all ages due to its chronic and recurrent nature (1–3). Uropathogenic *Escherichia coli* (UPEC) strains are thought to be the main etiologic agents associated with UTIs. These isolates originate from patients’ intestinal normal flora and lead to UTI when fecal *E. coli* colonizes the periurethral region (4). Consequently it forms both intra- and extracellular biofilm-like communities within the urinary bladder (1), which thwarts the host immune-mediated clearance and develops resistance against antimicrobial therapy that finally results in persistent infections that are difficult to eradicate.

There are several virulence factors associated with UPEC, e.g., fimbriae 1, hemolysis, serum resistance, hydrophobicity, but type 1 fimbriae accounts for more than 95% of the total *E. coli* virulence factor, causing UTI (5–7).

**Figure 1:**
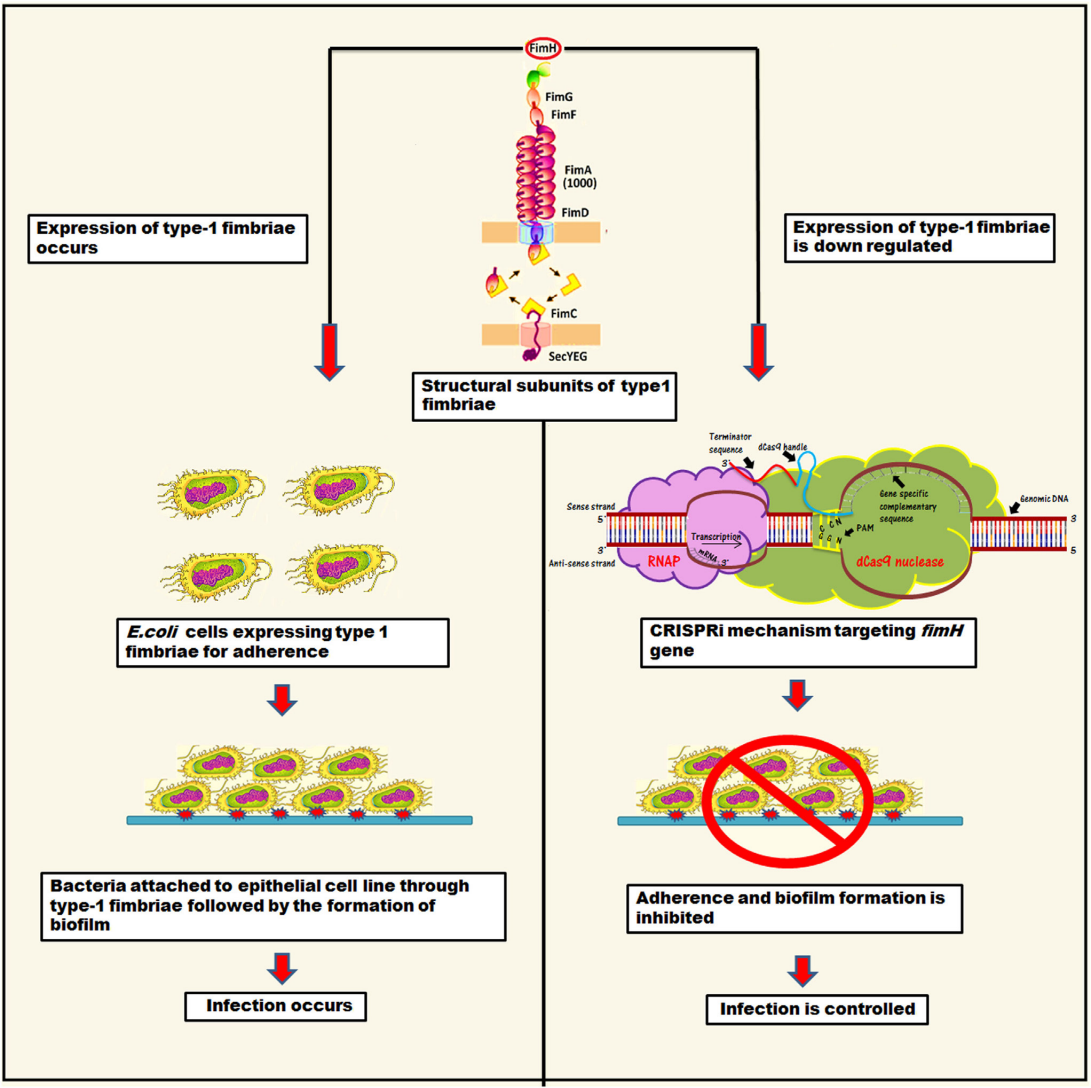
The representation of mechanistic approach of biofilm inhibition through CRISPRi for gene suppression of *fimH*.

Type 1 fimbriae are approximately 0.5–1.5 µm long structures, assembled through a chaperone-usher pathway (5, 8). They are also known as attachment pilus and are prevalent in gram negative bacteria (9, 10). Usually, bacteria utilize these structures to carry adhesions at their tip for attachment in order to obtain nutrients and withstand shear forces (10, 11). In *E. coli*, these type 1 fimbriae carry 30-kDa highly structural conserve adhesive subunit FimH, which is employed for mannose specific adherence (5, 11). FimH occurs in two alternative conformations depending on interaction of its two structural domains, i.e., lectin domain (LD having mannose binding pocket) and pillin domain (PD connecting FimH with other pillin minor subunits like FimG) (5, 12). LD and PD are connected *via* a short linker chain and on their close interaction LD exists in twisted and compressed conformation that leads to an opening of mannose binding pocket. Hence, as a result, close interaction between LD and PD leads to much lower affinity for mannose binding than in separated domain conformation (5). The binding of mannose to FimH is allosterically regulated that leads to shuffling between its two alternative conformations (5). Apart from FimH, the structural role of type 1 fimbriae is also governed by other subunits, namely FimA (main pillin subunit), FimG, and FimH (minor subunits) (8, 13–15). The mannose binding domain (LD) of the FimH is mainly responsible for adherence, which is the first step of infection. Hence, through fimbriae, bacteria attach to the substratum in order to withstand shear forces and absorb nutrients. The structural components of type 1 fimbriae are shown in Figure 1.

This study was planned to inhibit adhesion property of bacteria by knocking down its *fimH* gene that ultimately control UTI or other *E. coli* adherence associated infections. Hence, we designed CRISPRi (CRISPR interference), the gene perturbation technique to inhibit *fimH* gene expression. It tweaks the gene expression reversibly and accurately by hindering transcriptional machinery through lodging inactive or “dead” Cas9 at specific position (16, 17).

## Materials and Methods

### Bacterial Strain Collection and Characterization

Strain collection was performed from UTI cases, attending Jawaharlal Nehru Medical College & Hospital (JNMCH), Aligarh Muslim University (AMU) from October 2016 to March 2017. A total of 64 clean catch urine samples were collected from infected patient’s catheters in a wide mouth sterile container from the study subjects. Isolation was performed by a surface streak procedure on Luria Bertani (LB) broth (Himedia Labs, Mumbai, India) using calibrated loops for semiquantitative method and incubated aerobically at 37°C for 24 h, and those cultures which were negative after 24 h incubations, were further incubated for 48 h. A specimen was considered positive for UTI if a single organism was cultured at a concentration of ≥10^5^ cfu/ml. Out of 64 urine samples, 42 samples were found to be positive for UTI. Of 42, 26 samples were characterized to have *E. coli* isolates, confirmed through BD phoenix-100™ Automated Microbiology system using panel NMIC/ID-55.

For *fimH* detection a single colony on LB-plate from 26 remaining isolates were suspended in 100 µl of sterilized water and were incubated at 95°C for 10 min followed by centrifugation at 8,000 *g* for 10 min. Supernatant was used as template to perform colony PCR amplification under the condition, mentioned in supplementary data (Tables S1 and S2 in Supplementary Material) using appropriate primers. Among 26 isolates, 19 were reported to have *fimH*. Further detailed study was performed on one of the strains (AK-118) of 19 *fimH* positive strains.

### Culture and Growth Conditions

*Escherichia coli* clinical strain, AK-118 was used in this study. The strain was isolated from UTI patient, admitted in JNMCH AMU, Aligarh, India. The bacteria were cultured in LB broth (Himedia labs, Mumbai, India). The plasmids pdCas9 and pgRNA used in study, already mentioned in our earlier study (16). LB media supplemented with proper antibiotic (ampicillin 100 µg/ml and chloramphenicol 25 µg/ml) and inducer [anhydrotetracycline (aTc) 2 µM] was used, wherever needed. *E. coli* Top10 cells were used for transformation, while the cotransformation was performed in clinical strain. The microorganism were grown without (on agar) or with (in broth) shaking at 220 rpm at 37°C, overnight. Knockdown strain was supplemented with 2 µM aTc with their respective antibiotics, to suppress gene expression.

### Cloning and Construction of Knockdown Strain

The detailed protocol for cloning has already been mentioned in our earlier study (16). The complementary sequences, i.e., 20 bp region immediately following to 5′-CCN-3 [protospacer adjacent motif (PAM)] of *fimH* gene was commercially synthesized as a primers with additional 35 nt region of dCas9 handle (Table S1 in Supplementary Material). It was finally inserted through inverse PCR (Table S2 in Supplementary Material) in pgRNA (ampicillin resistant). The final PCR products were cleaned and ligated to give new sgRNA expressing plasmid pgRNA-FM to be ready for cotransformation along with pdCas9 (chloramphenicol resistant) in AK-118. The new knockdown strain was named as AK-FM1 for the record. The conditions used for PCR in this study were given in Table S2 in Supplementary Material.

### RT-PCR and mRNA Quantification

Extraction of total RNA was performed by Trizol method from respective strains grown in presence of inducer (2 µM aTc) and proper antibiotic concentration till log phase. Total RNA was treated with RNase-free DNase in order to remove DNA contamination and subsequently cDNA was prepared by high capacity cDNA Reverse Transcription Kits (Applied Biosystems, USA) according to manufacturer’s instruction. Quantification of mRNA was performed through comparative Ct method using SYBR green PCR master mix, along with 150 ng of cDNA sample with appropriate primers. The cycle was carried out at 95°C for 10 min, 95°C for 15 s, 60°C for 30 s, and finally 72°C for 30 s. The standard graphs for respective transcripts were observed using 16s rRNA as endogenous gene control.

### Mannose Sensitive Hemaggultination Assay on Guinea Pig Erythrocytes for Type 1 Fimbriae Expression

In our study, hemaggultination assay was performed on guinea pig erythrocytes with 1% mannose for type 1 fimbriae detection as described in previous study with the slight modification (18). A consent from institutional animal ethical committee of Interdisciplinary Biotechnology Unit (IBU), AMU (held on June 5, 2017, at IBU, AMU), was taken to perform this experiment. Bacteria were grown overnight in LB at 37°C for full fimbriation. The bacterial suspensions were adjusted to McFarland standard (0.5–0.6) in PBS or PBS/mannose 1% and serially diluted from 1:2 to 1:128 with same buffer in 96-well microtiter plate. 0.5% erythrocytes (in PBS or PBS/mannose 1%) was added to wells. Suspensions of bacteria and erythrocyte were incubated overnight at 4°C. All assays were conducted in triplicate. An *E. coli* ATCC strain (type 1 fimbriae positive) was used as a positive control.

### Morphological Studies on *fimH* Knockdown

Morphological study was performed using transmission electron microscopy using the standard TEM protocol to investigate the presence and absence of type 1 Fimbriae. Untreated and treated bacterial cultures were suspended using a centrifuge followed by washing with PBS (pH 7.4). Secondary fixation was done using gluteraldehyde (2.5%) and osmium tetroxide (1%) for 2–3 h at 4°C. Further Samples were dehydrated by ethanol and finally embedded in araldite CY212 (Taab, Aldermaston, UK) resin to make cell-pellet blocks. Ultrathin sections of cells stained with uranyl acetate and lead citrate were observed under the TEM (Model: JEM 2100, Jeol, Tokyo, Japan).

### Crystal Violet (CV) Assay

Biofilm formation was estimated by CV assay as used in our earlier study (19). Microtiter plates carrying secondary culture of treated and untreated bacterial cells were incubated at 37°C for 24 h, then medium was decanted and the planktonic cells were removed by gentle rinsing with sterile water. The adhered biofilms were stained for 15 min at room temperature with 200 µl of 0.1% CV dye and the bound dye was released from the cells by adding 98% ethanol and keeping the plates on shaker for 5 min. Biofilm quantification was done by measuring the optical density (OD) at 630 nm using a Bio-Rad iMark™ Microplate Reader, India.

### Cell Viability Assay (XTT Assay)

To assess metabolic activity and cellular viability, XTT assay was carried out as described earlier (19). XTT (1 mg/ml) was dissolved in filter sterilized PBS solution and menadione (0.4 mM) was freshly prepared in acetone. Fresh mixture of 20:1 volume of XTT and menadione was used. After 24 h of growth, biofilms were washed with 200 µl of PBS solution followed by the addition of XTT-menadione and PBS solution to a volume of 42 and 158 µl in each well, respectively, and kept at 37°C in dark for 4 h. After 4 h, the intensity of orange color formazan compound was measured, that quantify the ability of metabolically active sessile cells to reduce tetrazolium salt (2,3-bis(2-methoxy-4-nitro-5-sulfo-phenyl)-2H-tetrazolium-5-carboxanilide), using microtiter plate reader at 490 nm.

### Adherence Study on Human Intestinal Mucus Visualized through Confocal Microscopy

Confocal microscopy was performed to visualize the adherence property of bacteria with the human intestinal mucus. For that a resected human intestine tissue was taken as a source of intestinal mucus, which was approved by the Institutional ethical committee of IBU, AMU held on June 3, 2017, at IBU (AMU) also an informed written consent was taken from patient which remained confidential as per the protocol, established in our institution. The healthy part of large intestine was taken from the patient of diverticulitis and processed within 20 min of sample collected on ice. Mucus was isolated from the sample after washing it with PBS containing 0.01% gelatin by gently scraping through rubber spatula and collected in HEPES-HANKS buffer (10 mmol/L HEPES; pH 7.4). The collected mucus was centrifuged (13,000 *g*, 10 min) to remove cell debris and bacteria and stored at −20°C for further use after measuring protein content.

As described elsewhere (20), in adhesion assay mucus was diluted to the concentration 0.5 mg/ml with HEPES-HANKS buffer and 100 µg of it was immobilized directly on confocal dishes and incubates at 4°C overnight. The overnight grown bacteria after washing with PBS and adjustment of OD 0.5 at 600 nm, were stained with DAPI (355 nm wavelength for excitation and 460 nm wavelength for emission) to a final concentration of 0.2 µg/ml (DAPI) by incubating it for 30 min on mild shaking at room temperature. Finally the bacterial cells were washed three times with PBS and added to a volume of 100 µl into confocal dishes. Bacteria were allowed to adhere at 37°C for 1 h and wells were washed with HEPES-HANKS buffer to remove non adherent bacteria. The adherent bacterial cells were observed by Zeiss LSM 780 (Germany) confocal laser scanning microscope equipped with argon and He-Ne laser.

### Fluorescence Spectroscopic Studies of Adherent Bacterial Cells with Human Intestinal Mucus

To perform fluorescence spectroscopic studies the above mentioned adherent bacteria were released and lysed with 1% SDS-0.1 M NaOH by incubation at 60°C. The florescence quenching of released stained bacteria (DAPI stained) were determined by using Shimadzu RF-5301PC spectrofluorometer (Shimadzu Corporation, Kyoto, Japan) equipped with a thermostatically controlled cell holder. The filters used were 355 nm for excitation and 460 nm for emission.

### Statistical Analysis

The results were reported as mean ± SD. All experiments were conducted in triplicate. Further each experiment was compared with control for analyzing Student’s *t*-test, two-tailed hypothesis (*p < 0.05, *t*-test, two sided) (**p < 0.005, *t*-test, two sided).

## Results

### CRISPRi-Mediated *fimH* Gene Inhibition

**Figure 2:**
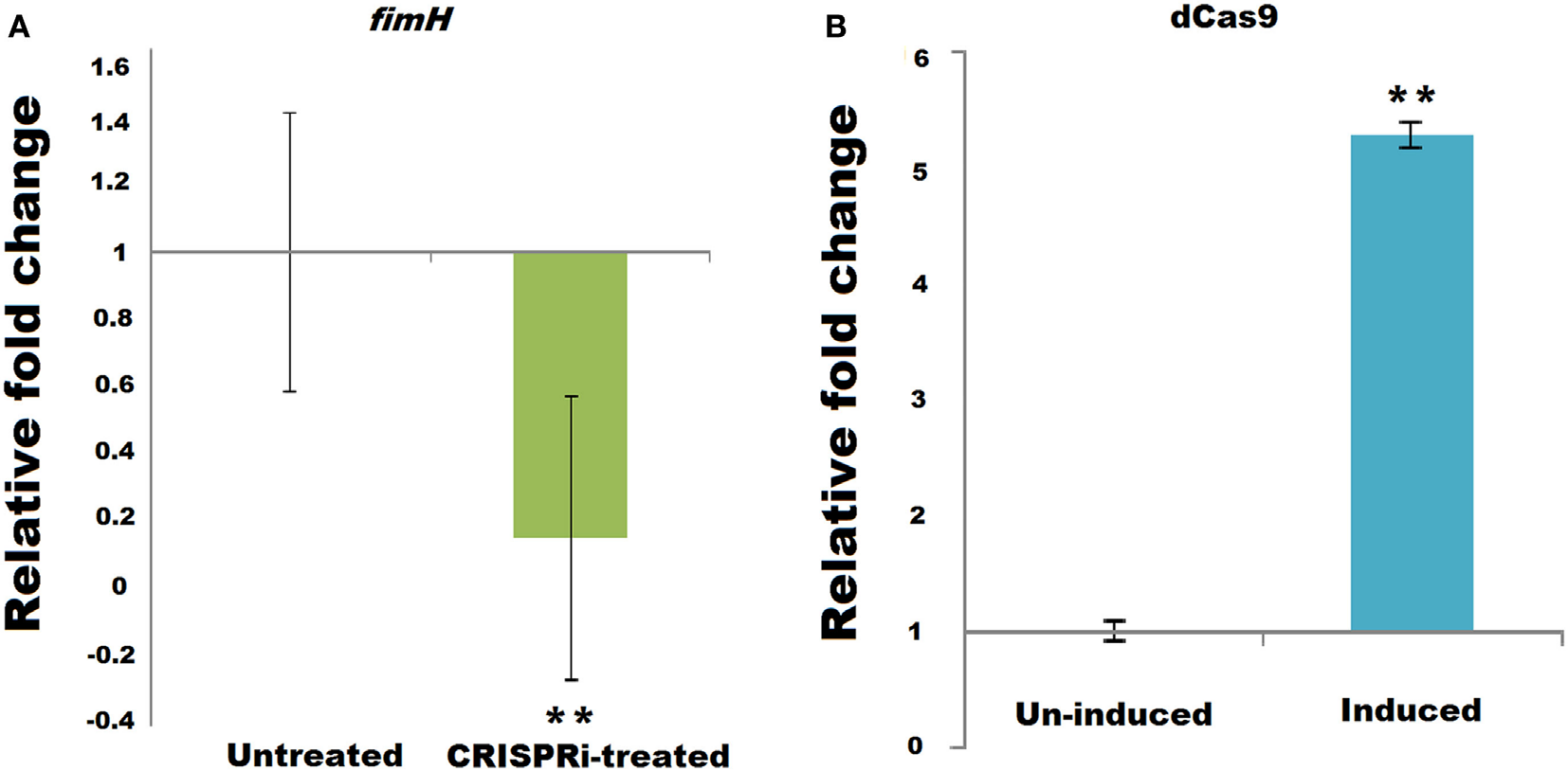
Relative qRT-PCR data of **(A)** *fimH* and **(B)** dCas9 expression.

Successful implication of CRISPRi was assayed through *fimH* mRNA quantification using relative qRT-PCR, taking 16s rRNA gene as endogenos control. Figure 2A corresponds to the relative qRT-PCR data of *fimH* which showed the downregulation of *fimH* gene in treated condition. Taking value of relative fold change in untreated condition as 1 ± 0.414, the value of relative fold change in treated condition was observed to be 0.1560 ± 0.419, i.e., 84.39% of downregulation was observed with *P*-value < 0.005. Figure 2B corresponds to relative qRT-PCR data of dCas9 nuclease in strain AK-118 and proved its successful expression in this strain under induced condition with relative fold change values of 5.2986 ± 0.099 as compare to uninduced condition values 1 ± 0.080 (*p*-value < 0.005).

### Expression of Type 1 Fimbriae

**Figure 3:**
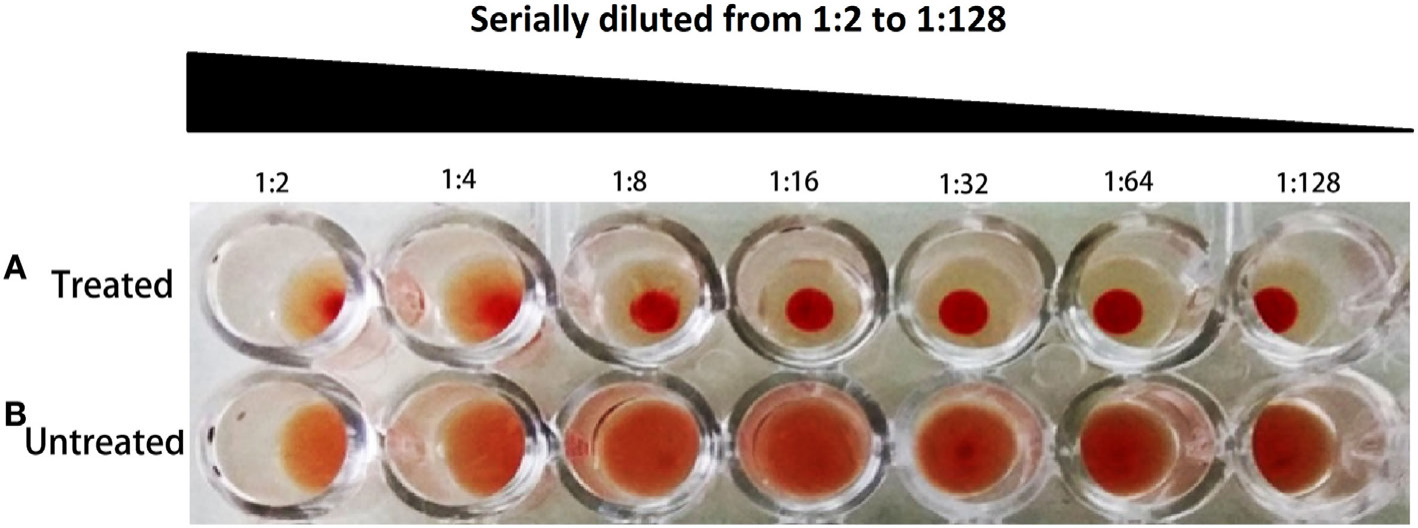
Hemaggultination assay on guinea pig erythrocytes. Row **(A)** treated and row **(B)** untreated.

**Figure 4:**
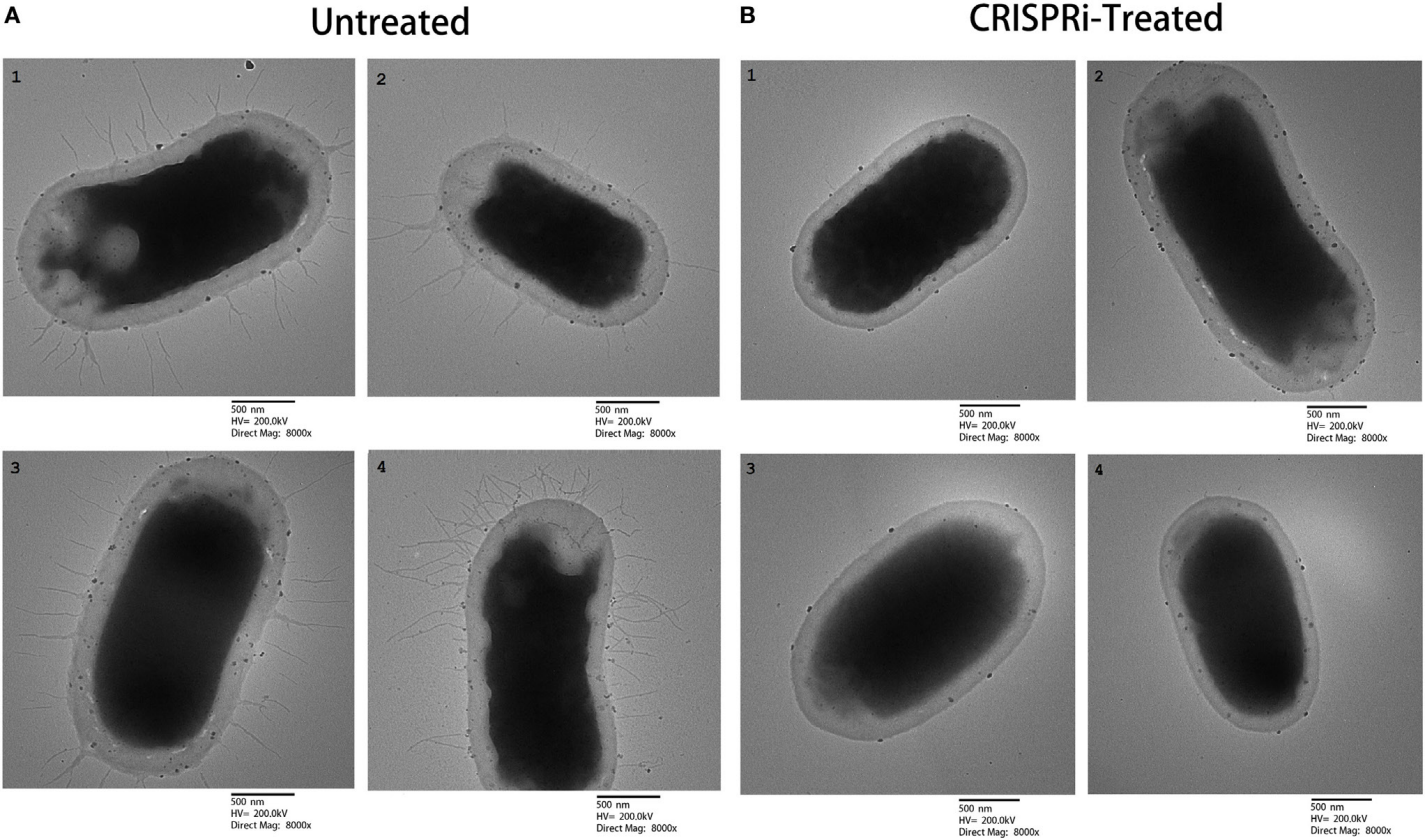
TEM images from four different visual fields. **(A)** Untreated and **(B)** CRISPRi treated.

The effect of *fimH* gene suppression for type 1 fimbriae expression was assayed by Mannose sensitive hemaggultination assay on guinea pig erythrocytes and TEM studies. In Figure 3, row A and row B corresponds to AK-FM1 under treated and untreated conditions, respectively. In row A, a least agglutination was observed in first two wells followed by formation of blood button in the center of the well on serial dilution, while in row B, agglutination of guinea pig erythrocytes were clearly seen in almost all wells. Figure 4 shows the TEM images of AK-FM1 single bacterium in untreated (Figure 4A) and treated (Figure 4B) conditions taken from four different visual fields. In control the fimbriae appendages were present on bacterial surface due to the expression of type 1 fimbriae, while in treated cells nearly smooth bacterial surface was observed.

### Quantification of Biofilm and Cell Viability

**Figure 5:**
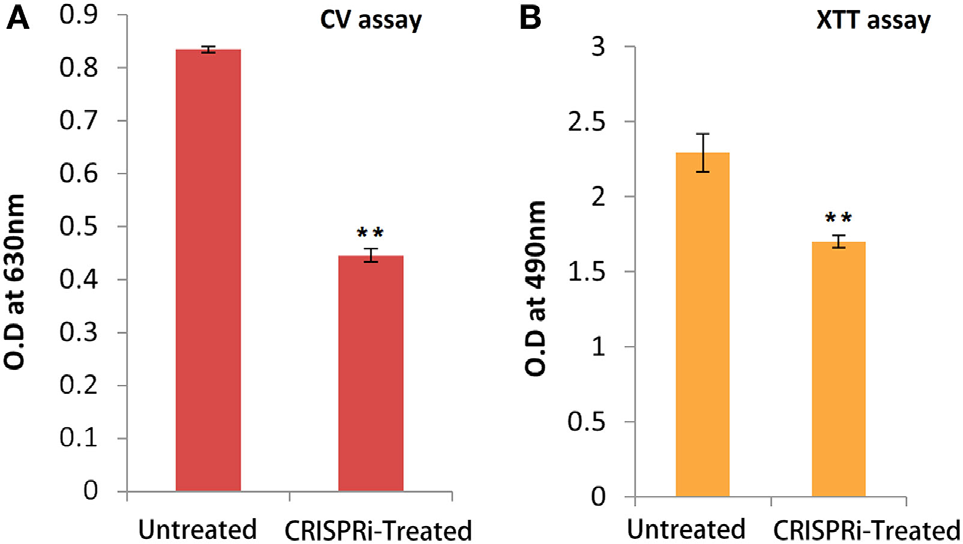
**(A)** Quantification of biofilm through crystal violet (CV) assay. **(B)** Cell viability assay (XTT assay). The data represent an average of triplicate experiments ± SD (*p < 0.05, *t*-test, two sided) (**p < 0.005, *t*-test, two sided).

The biofilm forming tendency of AK-FM1 after CRISPRi treatment was preliminary investigated by CV assay as shown in Figure 5A. The observed value of CV assay in treated sample was 0.445 ± 0.012 (*p*-value < 0.005) as compared to untreated sample value 0.834 ± 0.006 (*p*-value < 0.005). The downregulation of *fimH* gene in knockdown strain, AK-FM1 (treated) showed the dramatic decrease in biofilm formation as quantified by CV assay. The metabolic activity and cell viability of AK-FM1 in treated condition was recorded to be 74.06% with the values of 1.69 ± 0.040 and 2.29 ± 0.126 (*p*-value < 0.005) in treated and untreated conditions, respectively, as quantified by the converted amount of XTT in XTT reduction assay (Figure 5B).

### Effect on Bacterial Adherence

**Figure 6:**
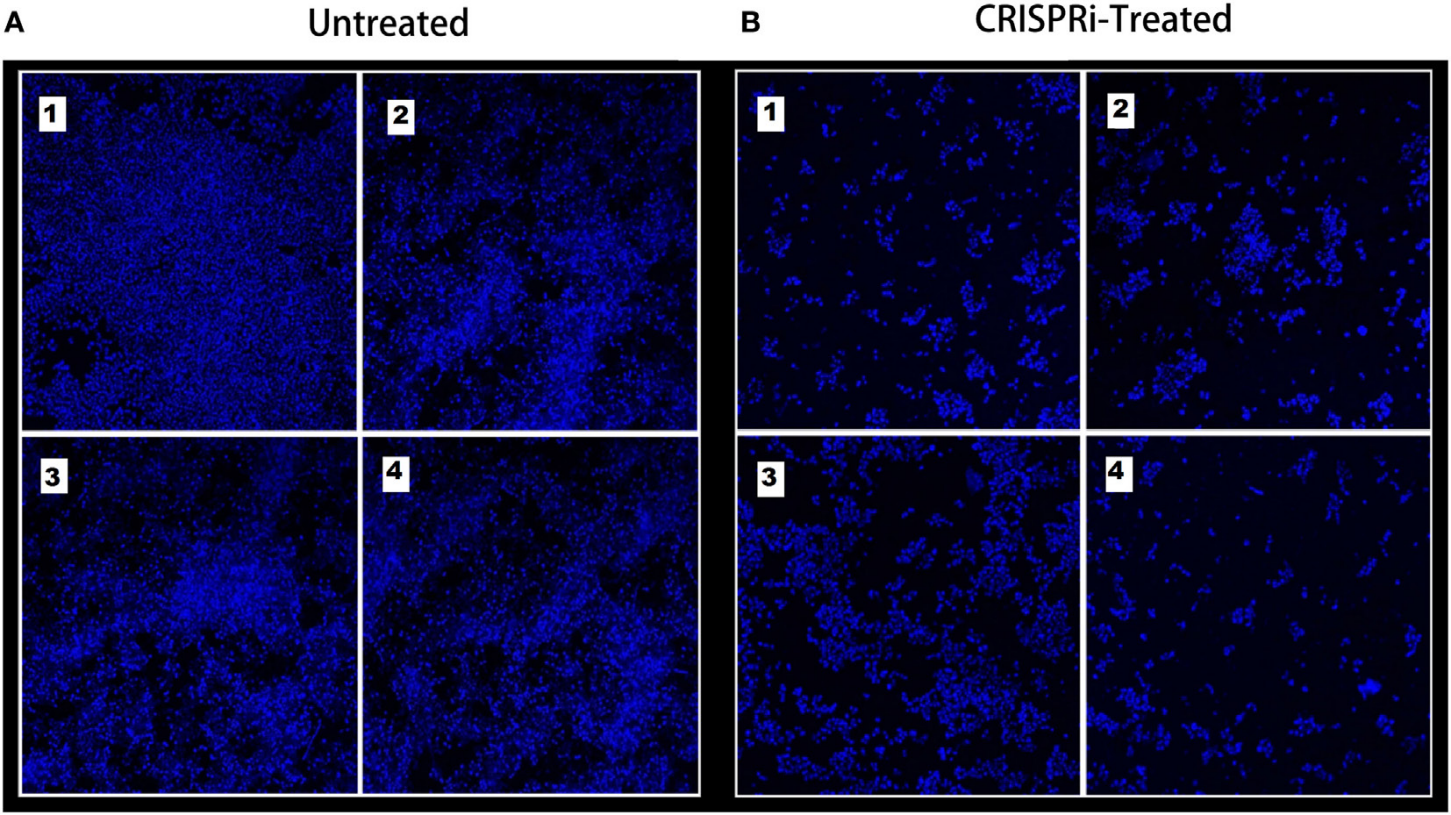
Adherence assay through confocal microscopy, images are taken from four different visual fields at 63× magnification. **(A)** Untreated and **(B)** CRISPRi treated.

**Figure 7:**
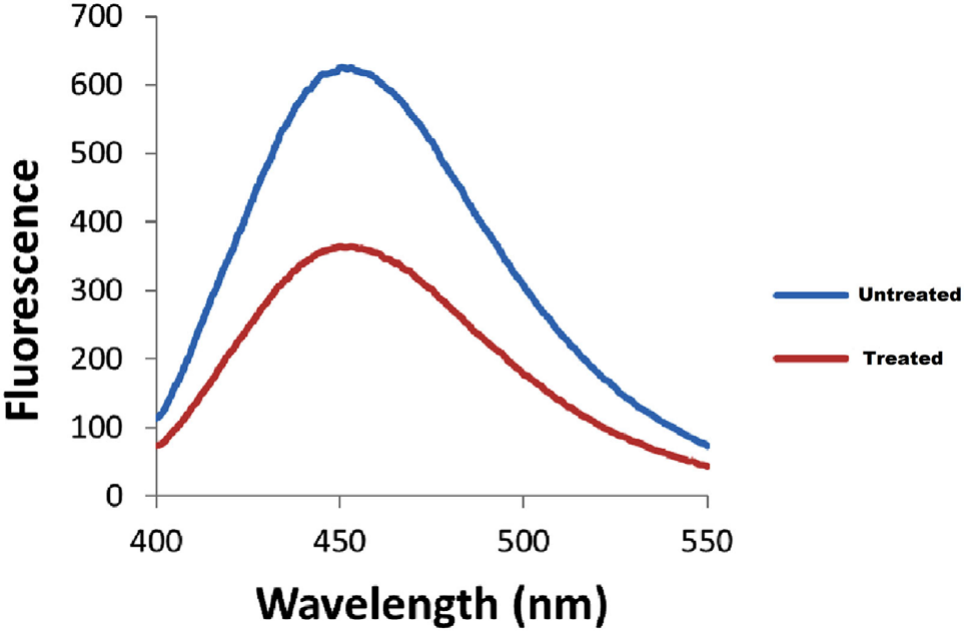
Fluorescence spectra of adherent bacterial cells. Quenching is observed in treated samples.

The bacterial adherence level on human intestinal mucus after CRISPRi-mediated *fimH* gene suppression was visualized through confocal microscopy. Figures 6A,B correspond to confocal microscopy images of untreated and treated samples of AK-FM1 taken from four different visual fields, respectively. The difference in the intensity of blue color due to DAPI stained adherent bacterial cells in untreated and treated samples, indicated the noticeable reduction in the adherence property of a bacteria after CRISPRi treatment. The same result came out from fluorescence spectroscopic studies where the quenching was observed in treated samples due to decrease in number of bacterial cells adhered to human intestinal mucus (Figure 7).

## Discussion

The main cause of UTI is adherence of bacterial aggregates on the inner lining of urinary tract or inner part of catheters, consequently leading to the formation of resistant biofilms, which are difficult to be eradicated. About 10–15% of patients develop UTI on short term catheterization mainly due to the bacterial growth and biofilm formation in its inner part (4). Among all bacterial strains, UPEC strains are responsible for about 50 and 80% of hospital and community acquired UTI infections, respectively (21). Such a high percentage of UPEC strains in these infections are due to the presence of different virulence factors like adhesions, hydrophobicity, hemolysin, serum resistance, drug resistance, gelatinase production, etc. Among all adhesions, type 1 fimbriae is thought to be the most important virulence factor that may result in worsening of UTIs (21). In UTI, FimH is a major detriment of type 1 fimbriae for adherence, because of the presence of high tropism for urinary tract receptors. It was further explained in detail that FimH contains mannose binding pocket that recognizes mannose containing glycoprotein receptors present on the host cell surface. In case of UTI, the apical surface of urinary bladders bears integral membrane uroplakin 1a that acts as a main receptor for FimH (22). Hence, the epidemiological typing of UPEC was performed through single-nucleotide polymorphism analysis of FimH (23, 24). Keeping this in mind, we have designed a CRISPRi based gene editing study against *fimH* gene to inhibit bacterial adherence and biofilm formation in UTI cases.

CRISPRi is a technique of genetic perturbation at transcriptional level that enable gene repression or activation. Although there are some potential limitations with the use of this technique for, e.g., requirement of PAM sequence within the target site, off-targeting, etc., but this can be solved by using different Cas9 homologs with longer PAM (25). Moreover, this technique has shown its advantage over other gene manipulating techniques because of its successful delivery through cell-mediated transfer in bacterial system (26). Our study was planned to inhibit *fimH*, the gene responsible for mannose receptor adhesion and type 1 fimbriae polymerization initiation. This is a lead study or a proof of concept for future implementation of this technology/approach to inhibit biofilm formation as it can be said that mixing this CRISPRi modified strain with natural population of *E. coli* (or preexisting biofilm) would hinder new biofilm formation and adherence of bacteria that will ultimately control the infection. The novelty of using CRISPRi in this study is that, in future this could be a potential approach to control infections like UPEC in clinical settings by directly delivering the CRISPRi modified cells at the site of infection through natural conjugation.

The experimental setup was established by designing CRISPRi to knockdown *fimH*. The effect of *fimH* downregulation on type 1 fimbriae was then checked through mannose-sensitive hemagglutination assay and TEM microscopic studies. The possible reason behind these observations is that, being an important component, FimH adhesion regulates the synthesis of type 1 fimbriae *via* chaperone usher pathway (27). It stimulates and initiates the polymerization of type 1 fimbriae through interacting FimD (28). For quantification of biofilm formation in these strains, CV assay was performed, where noticeable reduction was observed because of the inhibition of type 1 fimbriae that guides the first stage of biofilm formation. Finally, to check the adherence level of *fimH* knockdown strain, a study was performed on human intestinal mucus and the adhered bacteria were visualized through confocal microscopy. The dense layer of bacteria adhered on mucus was observed in untreated sample as compared to treated. The density of cells was reduced to very low level due to the absence of type 1 fimbriae expressions that abrogate the adherence property of bacteria to a large extent. These results highlight a new era where genomic manipulations are no more a hindrance to experiments and moreover they pave the way toward the major discoveries in science, with applications in all areas of biotechnology and human therapeutics.

## Conclusion

We conclude the role of CRISPRi in reducing the fimbriae of UPEC by suppressing the expression of *fimH*. The study has opened the new vistas to treat UTI by inhibiting biofilm formation of UPEC. Therefore, this approach has been proved as one of the potential strategies to control biofilm-mediated UTI. Hence, we further propose this approach to be validated and implemented *in vivo* infection model as to proof its use in UTI treatment therapy.

## Ethics Statement

Approved by the Institutional ethical committee of Interdisciplinary Biotechnology Unit (IBU), Aligarh Muslim University (AMU) held on 03-06-2017 at IBU (AMU) also an informed written consent was taken from patient who remained confidential as per the protocol, established in our institution.

## Author Contributions

AK designed experiments, analyzed data, and wrote the manuscript. AZ performed experiments and wrote the manuscript. NA performed experiments.

## Conflict of Interest Statement

The authors declare that the research was conducted in the absence of any commercial or financial relationships that could be construed as a potential conflict of interest. The reviewer CF and handling editor declared their shared affiliation.

## References

[1] Blango MG Mulvey MA. Persistence of uropathogenic *Escherichia coli* in the face of multiple antibiotics. Antimicrob Agents Chemother (2010) 54:1855–63. 10.1128/AAC.00014-10 20231390 PMC2863638

[2] Kunin CM Urinary tract infections in females. Clin Infect Dis (1994) 18:1–12. 10.1093/clinids/18.1.1 8054415

[3] Soto SM Importance of biofilms in urinary tract infections: new therapeutic approaches. Adv Biol (2014) 2014:13 10.1155/2014/543974

[4] Mittal S Sharma M Chaudhary U. Biofilm and multidrug resistance in uropathogenic *Escherichia coli*. Pathog Glob Health (2015) 109:26–9. 10.1179/2047773215Y.0000000001 25605466 PMC4445292

[5] Tchesnokova V Aprikian P Kisiela D Gowey S Korotkova N Thomas W Type 1 fimbrial adhesin FimH elicits an immune response that enhances cell adhesion of *Escherichia coli*. Infect Immun (2011) 79:3895–904. 10.1128/IAI.05169-11 21768279 PMC3187269

[6] Johnson JR Stamm WE. Diagnosis and treatment of acute urinary tract infections. Infect Dis Clin North Am (1987) 1:773–91. 3333658

[7] Warren JW Clinical Presentations and Epidemiology of Urinary Tract Infections. Urinary Tract Infections: Molecular Pathogenesis and Clinical Management. Washington, DC: ASM Press (1996). p. 3–27.

[8] Nishiyama M Ishikawa T Rechsteiner H Glockshuber R. Reconstitution of pilus assembly reveals a bacterial outer membrane catalyst. Science (2008) 320:376–9. 10.1126/science.1154994 18369105

[9] Berne C Ducret A Hardy GG Brun YV Adhesions involved in attachment to abiotic surfaces by Gram-negative bacteria. Microbiol Spectr (2015) 3(4). 10.1128/microbiolspec.MB-0018-2015 PMC4566860 26350310

[10] Tripathi P Beaussart A Alsteens D Dupres V Claes I von Ossowski I Adhesion and nanomechanics of pili from the probiotic *Lactobacillus rhamnosus* GG. ACS Nano (2013) 7:3685–97. 10.1021/nn400705u 23531039

[11] Weissman SJ Chattopadhyay S Aprikian P Obata Yasuoka M Yarova Yarovaya Y Stapleton A Clonal analysis reveals high rate of structural mutations in fimbrial adhesions of extra intestinal pathogenic *Escherichia coli*. Mol Microbiol (2006) 59:975–88. 10.1111/j.1365-2958.2005.04985.x 16420365 PMC1380272

[12] Le Trong I Aprikian P Kidd BA Forero-Shelton M Tchesnokova V Rajagopal P Structural basis for mechanical force regulation of the adhesion FimH via finger trap-like β sheet twisting. Cell (2014) 141:645–55. 10.1016/j.cell.2010.03.038 PMC2905812 20478255

[13] Munera D Palomino C Fernández LÁ Specific residues in the N terminal domain of FimH stimulate type 1 fimbriae assembly in *Escherichia coli* following the initial binding of the adhesin to FimD usher. Mol Microbiol (2008) 69:911–25. 10.1111/j.1365-2958.2008.06325.x 18627459

[14] Antão EM Wieler LH Ewers C. Adhesive threads of extraintestinal pathogenic *Escherichia coli*. Gut Pathog (2009) 1:22. 10.1186/1757-4749-1-22 20003270 PMC2797515

[15] Costa TR Felisberto-Rodrigues C Meir A Prevost MS Redzej A Trokter M Secretion systems in Gram-negative bacteria: structural and mechanistic insights. Nat Rev Microbiol (2015) 13:343–59. 10.1038/nrmicro3456 25978706

[16] Zuberi A Misba L Khan AU. CRISPR Interference (CRISPRi) inhibition of luxS gene expression in *E. coli*: an approach to inhibit biofilm. Front Cell Infect Microbiol (2017) 7:214. 10.3389/fcimb.2017.00214 28603699 PMC5445563

[17] Qi LS Larson MH Gilbert LA Doudna JA Weissman JS Arkin AP Repurposing CRISPR as an RNA-guided platform for sequence-specific control of gene expression. Cell (2013) 152:1173–83. 10.1016/j.cell.2013.02.022 23452860 PMC3664290

[18] Borowsky L Corção G Cardoso M. Mannanoligosaccharide agglutination by *Salmonella enterica* strains isolated from carrier pigs. Braz J Microbiol (2009) 40:458–64. 10.1590/S1517-83822009000300007 24031388 PMC3768530

[19] Misba L Kulshrestha S Khan AU. Antibiofilm action of a toluidine blue O-silver nanoparticle conjugate on *Streptococcus mutans*: a mechanism of type I photodynamic therapy. Biofouling (2016) 32:313–28. 10.1080/08927014.2016.114189 26905507

[20] Vesterlund S Paltta J Karp M Ouwehand AC Measurement of bacterial adhesion—in vitro evaluation of different methods. J Microbiol Methods (2005) 28:225–33. 10.1016/j.mimet.2004.09.013 15590097

[21] Hojati Z Zamanzad B Hashemzadeh M Molaie R Gholipour A The FimH gene in uropathogenic *Escherichia coli* strains isolated from patients with urinary tract infection. Jundishapur J Microbiol (2015) 8(2):e17520 10.5812/jjm.17520 25825648 PMC4376967

[22] Wiles TJ Kulesus RR Mulvey MA. Origins and virulence mechanisms of uropathogenic *Escherichia coli*. Exp Mol Pathol (2008) 85:11–9. 10.1016/j.yexmp.2008.03.007 18482721 PMC2595135

[23] Dias RC Moreira BM Riley LW. Use of fimH single-nucleotide polymorphisms for strain typing of clinical isolates of *Escherichia coli* for epidemiologic investigation. J Clin Microbiol (2010) 48:483–8. 10.1128/JCM.01858-09 20018817 PMC2815601

[24] Tartof SY Solberg OD Riley LW. Genotypic analyses of uropathogenic *Escherichia coli* based on fimH single nucleotide polymorphisms (SNPs). J Med Microbiol (2007) 56:1363–9. 10.1099/jmm.0.47262-0 17893175

[25] Larson MH Gilbert LA Wang X Lim WA Weissman JS Qi LS CRISPR interference (CRISPRi) for sequence-specific control of gene expression. Nat Protoc (2013) 8:2180 10.1038/nprot.2013.132 24136345 PMC3922765

[26] Ji W Lee D Wong E Dadlani P Dinh D Huang V Specific gene repression by CRISPRi system transferred through bacterial conjugation. ACS Synth Biol (2014) 3:929–31. 10.1021/sb500036q 25409531 PMC4277763

[27] Saulino ET Bullitt E Hultgren SJ. Snapshots of usher-mediated protein secretion and ordered pilus assembly. Proc Natl Acad Sci U S A (2000) 97:9240–5. 10.1073/pnas.160070497 10908657 PMC16852

[28] Munera D Hultgren S Fernández LÁ Recognition of the N terminal lectin domain of FimH adhesin by the usher FimD is required for type 1 pilus biogenesis. Mol Microbiol (2007) 64:333–46. 10.1111/j.1365-2958.2007.05657.x 17378923

