# Supplementary material for: CRISPRi Induced Suppression of Fimbriae Gene (fimH) of a Uropathogenic Escherichia coli: An Approach to Inhibit Microbial Biofilms
Source: Front Immunol. 2017 Nov 13;8:1552. doi: 10.3389/fimmu.2017.01552 (PMC5694031; doi:10.3389/fimmu.2017.01552)
Supplement: Supplementary file 1 [file data_sheet_1.pdf]

**CRISPRi induced suppression of fimbriae gene (fimH) of a Uropathogenic *Escherichia coli* : a novel therapeutic approach against the battle between microbial biofilms and host immunity**

**Azna Zuberi, Nayeem Ahmad and Asad U Khan***

Medical Microbiology and Molecular Biology Lab.,Interdisciplinary Biotechnology Unit, Aligarh Muslim University, Aligarh – 202 002, India.

| Name | Sequence (5'-3') | Purpose |
|---|---|---|
| **FimH-F** | AATAGGGATAGCGGTACCATGTTTTAGAGCTAGAAATAGCAAGTTAAAATAAGGC | Forward primer used to Insert gene targeting sequence in pgRNA |
| **FimH-R** | ACTAGTATTATACCTAGGACTGAGCTAGC | Reverse primer used to Insert gene targeting sequence in pgRNA |
| **Colony-F** | GGGTTATTGTCTCATGAGCGGATACATATTTG | Forward primer for colony PCR |
| **Colony-R** | CGCGGCCTTTTTACGGTTC | Reverse primer for colony PCR |
| **FimH-rt-F** | GATGCGGGCAACTCGATT | Forward primer for FimH gene RT- PCR |
| **FimH-rt-R** | CGCCCTGTGCAGGTGAA | Reverse primer for FimH gene RT-PCR |
| **dCas9-rt-F** | CGGAAGCGACTCGTCTCAA | Forward primer for dCas9 RT- PCR |
| **dCas9-rt-F** | CAAATACGATTCTTCCGACGTGTA | Reverse primer for dCas9 RT-PCR |

**Table 1:Primers used in the study. The highlighted region shows the target *fimH* gene sequence.**

| PCR TYPE | CYCLE NUMBER | DENATURING | ANNEALING | EXTENTION |
|---|---|---|---|---|
| **INVERSE PCR** | 1 | 98□C, 30 s | | |
| | 2-26 | 98□C, 10 s | 62□C, 30 s | 72□C, 1 min |
| | 27 | | | 72□C, 5 min |
| **COLONY PCR** | 1 | 95□C, 3 min | | |
| | 2-31 | 95□C, 30 s | 62□C, 30 s | 72□C, 2 min |
| | 32 | | | 72□C, 5 min |
| **qRT-PCR** (*fimH* and dCas9) | 1 | 95□C, 10 min | | |
| | 2-39 | 95□C, 15 s | 60□C, 30s | 72□C, 30 sec |
| | 40 | | | 72□C, 7 min |

**Table 2: Conditions used in PCR.**
